# Standardised neonatal parenteral nutrition formulations – Australasian neonatal parenteral nutrition consensus update 2017

**DOI:** 10.1186/s12887-020-1958-9

**Published:** 2020-02-08

**Authors:** Srinivas Bolisetty, David Osborn, Tim Schindler, John Sinn, Girish Deshpande, Chee Sing Wong, Susan E. Jacobs, Nilkant Phad, Pramod Pharande, Rodney Tobiansky, Melissa Luig, Amit Trivedi, Joanne Mcintosh, Eszter Josza, Gillian Opie, Lyn Downe, Chad Andersen, Vineesh Bhatia, Prasanna Kumar, Katri Malinen, Pita Birch, Karen Simmer, Gemma McLeod, Suzanne Quader, Victor Samuel Rajadurai, Michael Patrick Hewson, Arun Nair, Megan Williams, Jing Xiao, Hari Ravindranathan, Roland Broadbent, Kei Lui

**Affiliations:** 1 Royal Hospital for Women, Locked Bag 2000, Randwick NSW, Sydney, 2031 Australia; 2 Conjoint Lecturer, University of New South Wales, Sydney, Australia; 3 Royal Prince Alfred Hospital, Sydney, Australia; 4 University of Sydney, Sydney, Australia; 5 Royal Hospital for Women, University of New South Wales, Sydney, Australia; 6 Royal North Shore Hospital, Sydney, Australia; 7 Nepean Hospital, Sydney, Australia; 8 Hospital Kuala Lumpur, Kuala Lumpur, Malaysia; 9 Deputy Clinical Director and Neonatal Paediatrician, The Royal Women’s Hospital, Parkville, Victoria Australia; 10 John Hunter Children’s Hospital, Newcastle, Australia; 11 Monash Children’s Hospital, Clayton, Victoria Australia; 12 Liverpool Hospital, Sydney, Australia; 13 Westmead Hospital, Sydney, Australia; 14 The Children’s Hospital at Westmead, Sydney, Australia; 15 Head and Neonatal Paediatrician, Mercy Hospital for Women, Heidelberg, Victoria Australia; 16 Head of Neonatology, Women’s and Children’s Hospital, North Adelaide, Australia; 17 Townsville Hospital, Townsville, Australia; 18 PGCert Clinical Education, PGDip Child Health (associate), Advanced Pharmacist, Townsville Hospital, Townsville, Australia; 19 Gold Coast University Hospital, Southport, Australia; 20 King Edward Memorial Hospital for Women, Subiaco, Australia; 21 King Edward Memorial and Princess Margaret Hospitals, Subiaco, Australia; 22 The Sydney Children’s Hospital Network, Sydney, Australia; 23 Department of Neonatology, KK Women’s and Children’s Hospital, Kampong Java, Singapore; 24 Wellington Hospital, Wellington South, New Zealand; 25 Waikato Hospital, Hamilton, New Zealand; 26 Nepean Hospital, Penrith, Australia; 27 Paediatric Intensivist, Sydney Children’s Hospital, Sydney, Australia; 28 Dunedin School of Medicine, Dunedin, New Zealand; 29 University of New South Wales, Sydney, Australia

**Keywords:** Parenteral nutrition, Neonate, Preterm, Standardisation, Consensus

## Abstract

**Background:**

The first consensus standardised neonatal parenteral nutrition formulations were implemented in many neonatal units in Australia in 2012. The current update involving 49 units from Australia, New Zealand, Singapore, Malaysia and India was conducted between September 2015 and December 2017 with the aim to review and update the 2012 formulations and guidelines.

**Methods:**

A systematic review of available evidence for each parenteral nutrient was undertaken and new standardised formulations and guidelines were developed.

**Results:**

Five existing preterm Amino acid-Dextrose formulations have been modified and two new concentrated Amino acid-Dextrose formulations added to optimise amino acid and nutrient intake according to gestation. Organic phosphate has replaced inorganic phosphate allowing for an increase in calcium and phosphate content, and acetate reduced. Lipid emulsions are unchanged, with both SMOFlipid (Fresenius Kabi, Australia) and ClinOleic (Baxter Healthcare, Australia) preparations included. The physicochemical compatibility and stability of all formulations have been tested and confirmed. Guidelines to standardise the parenteral nutrition clinical practice across facilities have also been developed.

**Conclusions:**

The 2017 PN formulations and guidelines developed by the 2017 Neonatal Parenteral Nutrition Consensus Group offer concise and practical instructions to clinicians on how to implement current and up-to-date evidence based PN to the NICU population.

## Background

Parenteral nutrition (PN) is an essential component in the management of many newborn infants, admitted to Newborn Intensive Care Units (NICUs). All New South Wales (NSW) NICUs and subsequently other Australian NICUs formed a PN Consensus Group in 2010. Formulations were standardised in July 2011 with further amendments in 2012 and 2013. The Consensus Group recommendations and the improved nutritional outcomes following the implementation of consensus formulations were published in 2014 [1, 2]. Although nutritional intakes improved significantly, amino acid targets were not achieved in extremely preterm infants.

### Consensus process

In February 2015, the group reconvened in Sydney, now comprising 49 tertiary and non-tertiary NICUs from Australia, New Zealand, Malaysia, Singapore and India who participated in the consensus meeting in-person or via video link. A survey was conducted to explore the clinical PN practice in each NICU [3]. Outcomes of 2010 consensus formulations were presented, gaps in knowledge identified, topics for updating the formulations prioritised with tasks distributed to participants.

In September 2017, delegates reconvened, evidence was reviewed and guidelines and formulations were updated. A systematic literature search was undertaken and detailed reports were developed for each component of PN. Levels of evidence (LOE) and grades of recommendation (GOR) were allocated according to National Health and Medical Research Council (NHMRC) criteria [4]. Physicochemical compatibility and stability of updated formulations were checked and confirmed compliant by a compounding pharmaceutical facility (Baxter Pharmaceuticals Pty Ltd). The new formulations were commissioned and released in March 2018. The updated PN guidelines are based on the majority consensus of the PN Consensus Group. Where good published evidence was unavailable, recommendations were discussed and, if necessary, voted upon. Time was allowed for thorough literature review, free and open discussion and an exchange of good practice tips among units. Each member of the group was given the opportunity to provide comments and suggestions to the consensus group for their consideration. They are written balancing the potential benefits of PN against associated risks. These practice guidelines do not account for every clinical situation, particularly for infants that are acutely unwell or unstable. The professional judgement of the health professional in these individual cases must take precedence.

### Outcomes of the consensus

#### Indications

The survey of Australian and New Zealand NICUs in 2015 demonstrated that 97% of extremely preterm infants weighing < 1000 g and 88% of very preterm infants 1000–1500 g are commenced on PN from day 1 of life, and 77% of NICUs administer PN to any infant > 1500 g who fails to establish enteral feeds by day 3–5 of life [3]. The current recommendation remains the same as the 2012 recommendation (Table 1).

**Table 1:** Summary of key recommendations

Key points	Level of evidence (LOE); Grade of recommendation (GOR)^3`^
Indication: Preterm Infants < 32 weeks and/or < 1500 g – PN should be commenced within the first 12 h of life (on admission).	Consensus
Indication: Infants at high risk of NEC (e.g. absent or reversed foetal umbilical artery flow, perinatal asphyxia) or with illness in whom establishment of enteral feeding is likely to be delayed by 3–5 days.	Consensus
Fluids: Starting parenteral fluid intake at 60 ml/kg/day with daily increase by 20–30 ml/kg/day to an average maximum of 150 ml/kg/day. Titrate to clinical need (urine output and specific gravity, weight, serum sodium).	LOE I GOR B
Energy: Minimal energy requirements are met with 50–60 kcal/kg/day, but 100–120 kcal/kg/day facilitate maximal protein accretion. A newborn infant receiving PN needs fewer calories (90–100 kcal/kg/day) than a newborn fed enterally because there is no energy lost in the stools and there is less thermogenesis.	Consensus
Dextrose: Maximal glucose oxidation has been reported in preterm infants to be 8.3 mg/kg per min (12 g/kg per day) and in term infants 13 mg/kg per min (18 g/kg per day). Carbohydrate provides 40–60% of total energy.	Consensus
Amino acids: (1) commence parenteral AA within the first 24 h of birth (LOE I, GOR C), (2) commence parenteral AA at 2 g/kg/day (LOE II, GOR C), and (3) incrementally increase amino acid infusions to a maximum 4 g/kg/day by day 3–5 of life in preterm neonates (LOE I, GOR C). The safety of (1) commencement parenteral AA in excess of 3 to 3.5 g/kg/day and (2) maintenance AA intake in excess of 4.5 g/kg/day has not been proven in clinical trials.	LOE I
Lipids: Commence lipids at 1 g/kg/day and increase by 1 g each day to 3 g/kg/day. If lipid infusion is increased in increments of 0.5 to 1 g/kg per day, it may be possible to monitor for hypertriglyceridaemia [triglycerides > 2.8 mmol/L]. Essential fatty acid deficiency occurs rapidly and can be prevented with introduction of as little as 0.5 to 1 g/kg/day of lipid infusion [linoleic acid]. Reduce but do not stop lipid infusion in the event of hypertriglyceridaemia.	Consensus
Sodium: Minimal sodium intake of approximately 1 mmol/kg/day on day 1 using a starter PN formulation. Standard formulations will gradually increase sodium to a maximum 4.6 mmol/kg/d in preterm and 3.4 mmol/kg/day in term infants at 135 ml/kg/day of PN.	LOE II, GOR C
Potassium: Minimal potassium intake using starter PN formulation, with an increase in standard formulations to a maximum 3.0 mmol/kg/day in preterm and 2.7 mmol/kg/day in term infants.	LOE III-2, GOR C
Acetate and chloride: First 3 mmol/kg/day of anion to be provided as chloride, next 3.5 mmol/kg/day of anion [reduced from 6 mmol/kg/day] to be provided as acetate and thereafter as chloride again.	Consensus
Calcium, phosphorus and magnesium: Parenteral Ca and P intakes to a maximum of 2.3 mmol/kg/day and 1.8 mmol/kg/day respectively. LOE II For Mg intake a minimum of 0.2 mmol/kg/day and maximum of 0.3 mmol/kg/day is appropriate for LBW infants. LOE 111–3	GOR C
Trace elements: Add zinc, selenium and iodine as individual trace elements to all AA/dextrose formulations. For those infants, who are on exclusive PN for more than 2 to 4 weeks with minimal enteral intake, other trace elements (copper, manganese and molybdenum) can be added to the formulations.	LOE IV, GOR C
Heparin: Heparin for peripherally placed percutaneous central venous catheters found a reduced risk of catheter occlusion.	LOE I, GOR C
Hanging time: 48 h for PN solution and lipid.	LOE II, GOR C
Route of administration: Peripherally inserted central catheters (PICC’s) should be used preferentially to provide central venous access in neonates receiving prolonged PN as PICC use results in improved nutrient intake, fewer insertion attempts and fewer extravasation injuries. Umbilical vessels can be used for PN. UVC compared to peripheral venous catheter reduces insertion attempts with no increase in risk of infection or necrotising enterocolitis.	LOE I GOR B
Cessation of PN: Amino acid/dextrose infusion: cease when infant tolerating 120 (to 140) mL/kg/day of enteral feeds. Lipid: halve infusion when infant tolerating 100 mL/kg/day enteral feeds and cease when tolerating 120 mL/kg/day enteral feeds.	Consensus

#### Energy

Parenteral calorie recommendations remain similar, including consideration of the updated 2014 American Academy of Pediatrics (AAP) Committee on Nutrition recommendation of a parenteral caloric intake of 90–115 kcal/kg/day in preterm infants [5] and the ESPGHAN 2005 recommendation of 90–110 kcal/kg/day for extremely low birth weight (ELBW) infants [6].

The 2017 updated starter PN solution infused at 60 ml/kg/day (33 kcal/kg/day) and new starter concentrated PN solution at 40 ml/kg/day and lipid emulsion at 1 g/kg/day (10 kcal/kg/day) provides approximately 43 kcal/kg/day. The standardised preterm PN solution infused at 135 ml/kg/day (70.2 kcal/kg/day) and new concentrated preterm PN at 100 ml/kg/day and lipid emulsion at 3 g/kg/day (30 kcal/kg/day) provides approximately 100 kcal/kg/day (Level of Evidence [LOE] II, Grade of Recommendation [GOR] B).

#### Fluids

The 2017 consensus agreement is similar to the 2012 recommendation: Standardised PN should be formulated to provide recommended nutrient intakes (RNI) in a total fluid intake of 150 ml/kg/day. This includes 135 ml/kg/day of AA/dextrose formulation and 15 ml/kg/day water in the 20% lipid emulsion. The 2012 agreement on starting fluid intake at 60 ml/kg/day with daily increase by 20–30 ml/kg/day to an average maximum of 150 ml/kg/day remains. Alternatively, for those units who opt for a total water intake below 150 ml/kg/day, or unwell babies with multiple non-protein intravenous infusions such as inotropes, opioid analgesics contributing to a significant proportion of fluid volume, the new concentrated PN solutions provide an adequate nutrient and mineral intake on a lower volume.

#### Amino acids

There is no definitive evidence about what dose of parenteral amino acid is appropriate and when to initiate parenteral AA supplementation in neonates. Delay in administering amino acids could result in a protein catabolic state and could impact on growth and development in preterm neonates. However, potential benefits of improved nitrogen balance, growth and infant health may be outweighed by the infant’s ability to utilise high intakes of parenteral amino acid, especially in the days after birth, resulting in high concentrations of amino acids, ammonia and urea, and an exacerbation of metabolic acidosis. The 2017 consensus agreement incorporates the findings of three new systematic reviews that evaluated the efficacy and safety of parenteral AA in preterm neonates [7–9].

The Consensus Group recommendations remained unchanged: (1) commence parenteral AA within the first 24 h of birth (LOE I, GOR C); (2) commence parenteral AA at 2 g/kg/day (LOE I, GOR C) and; (3) incrementally increase amino acid infusions to a maximum 4 g/kg/day by day 3–5 of life in preterm neonates (LOE I, GOR C). The Consensus Group considered the safety of (1) commencement parenteral AA in excess of 3 to 3.5 g/kg/day and (2) maintenance AA intake in excess of 4.5 g/kg/day has not been proven in clinical trials.

#### Carbohydrates

Consensus recommendations regarding carbohydrates remain unchanged. Proposed standard preterm and term PN formulations contain 10 and 12% dextrose respectively, providing 13.5 g/kg/day (9.4 mg/kg/min) and 17 g/kg/day (11.8 mg/kg/min) at 135 ml/kg/day respectively (LOE I, GOR C).

#### Lipids

The Consensus Group reconsidered the findings of two systematic reviews, which reported no benefit of introducing lipids before two to five days [10, 11]. As the composition of growth was not assessed in these studies, it is uncertain if increased energy intake derived from early lipid infusion is protective against induced protein catabolism in the preterm neonate. Further, it is possible to prevent essential fatty acid deficiency with introduction of as little as 0.5 to 1 g/kg/day of lipid (linoleic acid) infusion [10]. The Consensus Group agreed to commencement of parenteral lipid on day 1 of PN administration, particularly for extremely preterm neonates (LOE I, GOR C).

Several types of intravenous lipid emulsions (IVLE) are available for neonatal use. Recent systematic reviews have shown no statistically significant differences between IVLEs with respect to clinically important outcomes including mortality, growth, chronic lung disease, sepsis, severe ROP ≥ stage 3, and cholestasis by using any specific preparation in newborns [12–14] (LOE I, GOR C). The 2015 Survey revealed 67% of units in ANZ use both mixed 30% soybean oil/25% olive oil/30% medium-chain triglyceride oil/15% fish oil IVLE (SMOFlipid [Fresenius Kabi, Australia]) and mixed 80% olive oil/20% soybean oil IVLE (ClinOleic [Baxter Healthcare, Australia]) [3]. All units use water and fat soluble vitamins added to lipid emulsion. The consensus 2017 proposed both SMOFlipid and ClinOleic as suitable lipid preparations and anticipate individual units will take cost and waste minimisation into consideration when choosing the specific type of lipid preparation most suited to their needs.

All lipid emulsions with added fat and water soluble vitamins discussed above are formulated to provide 1 g of lipid (SMOFlipid or ClicOleic) in 6 mL of lipid emulsion with vitamins (Soluvit N and Vitalipid N Infant). Thus, 1 g lipid/kg/day equates to 6 mL lipid emulsion with vitamins/kg/d, which equates to an energy intake of 10 kcal/kg/day. Similarly, 2 g lipid/kg/day equates to 12 ml/kg/day lipid emulsion with vitamins and 3 g lipid/kg/day equates to 18 ml/kg/day lipid emulsion with vitamins. There are variations in terms of starting dose of lipid emulsions. However, a starting dose 1 g/kg/day was safely tolerated in most clinical trials. The consensus agreed to commence lipids at 1 g/kg/day. There is no evidence that gradual increments in the infusion rate of lipids improve fat tolerance. The majority consensus was to increase by 1 g/kg (6 mL/kg) each day to 3 g/kg/day (18 mL/kg/day). If lipid infusion is increased in increments of 0.5 to 1.0 g/kg/day, it may be possible to monitor for hypertriglyceridemia [6]. The lipid emulsions contain 80% water (6 mL lipid emulsion with vitamins contains 5 mL water; 12 mL lipid emulsion with vitamins contains 10 mL water; 18 mL lipid emulsion with vitamins contains 15 mL water). In view of benefits associated with restricted fluid intake [15], the group proposed to include the water content of lipid infusions in the total fluid intake, which equates to 15 ml/kg/day of water when the lipid intake reaches 3 g/kg/day (LOE I, GOR B).

#### Sodium, potassium and chloride

The 2017 consensus reaffirmed the consensus reached in 2012, agreeing to minimal sodium intake of approximately 1 mmol/kg/d on day 1 using a starter PN formulation. Sodium in starter formulation is a component of organic phosphate (sodium glycerophosphate which contains 2 mmol Na per mmol of phosphate) and can only be altered by replacing with other sodium free nutrients. The currently designed starter and standard formulations will increase sodium from 1 mmol/kg/day on day 1 (starter formulation) to a maximum 4.6 mmol/kg/day in preterm infants and 3.4 mmol/kg/day in term infants at 135 ml/kg/day (standard formulations) (LOE II, GOR C). The new starter concentrated PN solutions will gradually increase sodium from 1.2 mmol/kg/day on day 1 in the starter concentrated PN to a maximum 5.0 mmol/kg/day in the concentrated preterm PN in extremely preterm infants. A Cochrane review assessing the benefits and harms of higher versus lower sodium intakes in preterm infants is currently underway [16].

The Consensus Group agreed on minimal potassium intake using starter PN formulation, with an increase in standard formulations to a maximum 3.0 mmol/kg/d in preterm and 2.7 mmol/kg/day in term infants (LOE III-2, GOR C). This is unchanged from the previous consensus and complies with AAP and ESPGHAN 2005 recommendations [5, 6].

With respect to chloride, the 2013 consensus was to adopt a trial recommendation that the first 3 mmol/kg/day of anion is provided as chloride, next 6 mmol/kg/day of anion is provided as acetate and thereafter as chloride again [17]. However, there have been concerns about hypercarbia in some infants using 2013 formulations. Moreover, 2017 consensus formulations contain organic phosphate (sodium glycerophosphate) resulting in an increase of pH of the formulations. Therefore, the 2017 consensus is to reduce the acetate content to a maximum of 3.5 mmol/kg/day in the updated standard formulations (LOE II, GOR C).

#### Calcium, phosphorus and magnesium

There is a wide range in the recommended doses of calcium (Ca) and phosphorus (P) delivered by PN in preterm infants. American Academy of Pediatrics 2014 recommends 1.5–2.0 mmol/kg/day of Ca and 1.5–1.9 mmol/kg/day of phosphorus whereas ESPGHAN 2005 recommends 1.3–3 mmol/kg/day of Ca and 1.0–2.3 mmol/kg/day of P, with optimal Ca:P ratio between 1.3–1.7 [5, 6]. However, these recommendations are based on in-utero accretion rates. While a number of clinical studies evaluated the efficacy and safety of various parenteral intakes of Ca and P in preterm infants, none of them tested the ESPGHAN recommended maximum intake levels of 3 mmol/kg/day of Ca and 2.3 mmol/kg/day of P in preterm neonates, so the safety of this level of intake is unreported [18–21]. One RCT used inorganic phosphate for an estimated maximum P intake 2.6 mmol/kg/day with a maximal Ca intake 2 mmol/kg/day [20].

Until recently, only inorganic phosphate has been registered by Therapeutics Goods Administration Australia and higher intakes of calcium and phosphate intravenously were not possible while maintaining compatibility to prevent calcium-phosphate precipitation [20, 21]. Recently, organic phosphate in the form of sodium glycerophosphate has been registered. Substitution of inorganic phosphate by organic phosphate improves physicochemical compatibility with trial evidence reported increased mineral intake and mineral retention [19].

Due to a paucity of studies on the safety of maximum parenteral RDIs for Ca and P and the availability of organic phosphate with improved physicochemical stability, the 2017 consensus increased the parenteral Ca and P intakes to a maximum of 2.3 mmol/kg/day and 1.8 mmol/kg/day respectively. Furthermore, there is additional 0.19 mmol/kg/day P from ClinOleic or SMOFLipid at 3 g/kg/day.

The 2017 consensus regarding magnesium (Mg) is unchanged. A minimum Mg intake of 0.2 mmol/kg/day and maximum 0.3 mmol/kg/day is considered appropriate for LBW infants. (LOE III-3, GOR C).

#### Vitamins

The lipid emulsion contains water and fat soluble vitamins (Soluvit N and Vitalipid N Infant [Fresenius Kabi, Australia]). There was no change with respect to vitamins in the 2017 consensus.

#### Trace elements

In the absence of a formulation with the optimal mixture of trace elements, the 2012 consensus was to add zinc, selenium and iodine as individual trace elements to all AA/dextrose formulations. Trace elements were not added to the starter formulation due to physicochemical compatibility concerns. The 2018 formulations contain organic phosphate allowing addition of trace elements in all formulations including the starter formulation without any physicochemical instability. Other trace elements, including copper, manganese and molybdenum, can be added to the formulations in infants who are on exclusive PN for more than 2–4 weeks with minimal enteral intake.

#### Heparin

Our previous and current consensus is to add heparin 0.5 IU/ml to AA/dextrose formulations (LOE I, GOR C) and remains the same.

#### Physicochemical stability

Physicochemical stability of the latest formulations have been tested and confirmed to be stable for up to 61 days at 2–8 °C and 5 days at below 25 °C (Baxter Healthcare, Australia).

#### Hanging time

The majority consensus 2017 recommended a hanging time of 48 h for PN solution. Due to concerns of microbial contamination with longer hanging times [22–24], a range of 24–48 h hanging time was agreed for lipid infusions (LOE II, GOR C).

#### Route of administration

##### Umbilical catheters

In neonates, umbilical vessels can be used for PN [6]. Umbilical venous catheters compared to peripheral venous catheter reduces insertion attempts with no increase in risk of infection or necrotising enterocolitis [25]. The risk of complication may increase if umbilical venous catheters are left in place for more than 14 days [26, 27].

##### Central cannula

Peripherally inserted central catheters (PICCs) should be used preferentially to provide central venous access in neonates receiving prolonged PN as PICC use results in improved nutrient intake and fewer insertion attempts [6, 28].

##### Peripheral cannula

As phlebitis of peripheral veins were reported when the osmolality of the intravenous solution exceeded 600 mOsm [29], peripheral veins have been recommended for short term venous access [6, 29]. The group has developed a peripheral preterm PN solution (see below) with reduced mineral content that can be used to run through the peripheral cannula for short periods of time, although there is limited evidence for the role of minerals in the development of tissue injury. Although extravasation injury occurs in up to 10% of infants managed only with peripheral infusion of PN [28], it is unclear if the risk of peripheral PN is greater than the risk of peripheral crystalloid infusion.

#### Osmolality

The formulations that have been developed as a result of the 2017 consensus are predominantly intended for administration via a central vein. American Society of Parenteral and Enteral Nutrition (ASPEN) recommends the osmolarity of peripheral parenteral nutrition solutions be limited to 900 mOsm/L to lower the risk of phlebitis due to infiltration based on a study that evaluated the feasibility of infusing a 900 mOsm/L solution through peripheral veins in 15 adult participants [30]. A prospective study reported that administration of PN with an osmolarity ≤1000 mOsm/L resulted in an 8% (15 of 181) incidence of extravasation/phlebitis, whereas peripheral administration of PN with osmolarity > 1000 mOsm/L resulted in a 30% (40 of 134) incidence of extravasation/phlebitis, suggesting that peripheral administration of PN in neonates should be limited to 1000 mOsm/L. [31] Another retrospective, matched-cohort study that included 151 neonates found that administration of PN with osmolarity > 1000 mOsm/L vs ≤1000 mOsm/L significantly increased infiltration (17% vs 7%; odds ratio [OR], 2.47; 95% confidence interval [CI], 1.24–4.94; *P* = .01) and the combined composite end point of phlebitis or infiltration (45% vs 34%; OR, 1.65; 95% CI, 1.07–2.54; *P* = .02). In multivariate analysis, osmolarity > 1000 mOsm/L vs ≤1000 mOsm/L was an independent risk factor for developing complications (OR, 1.67; 95% CI, 1.08–2.52; *P* = .02) [32].

The 2017 Consensus Group concluded that PN solutions with osmolality below 1000 mosm/L can be administered peripherally for short term use provided that close monitoring of the IV site for any extravasation/phlebitis is followed. In view of the dearth of evidence, the group agreed to continue the peripheral PN formulation in case of concerns regarding the amount of calcium infused through peripheral veins.

#### PN in late preterm (34^+0^to 36^+ 6^ weeks) and term neonates

There is paucity of data on the efficacy and safety of PN in this age group. Two small studies enrolled late preterm and term neonates, but neither reported on major clinical outcomes [33, 34]. PN is widely used in Australian facilities in late preterm and term neonates who are not enterally fed. The 2017 Consensus Group followed the human milk approach to develop the PN formulations for this group and nutrient intake estimates are based on the average composition and intake of human milk [35].

#### Updated consensus formulations

Table 1 provides the summary of recommendations and strength of evidence for recommendations. Based on these recommendations, the 2017 group developed 8 AA/dextrose formulations and 2 lipid formulations in different volumes. Six of these AA/dextrose formulations are the improved versions of 2012 formulations. These are summarised in Table 2. Two new AA/dextrose formulations have been proposed and the contents and indications of these formulations are shown in Tables 3-4. Suggested lipid formulations are shown in Table 5. Additional file 1: Tables S1 and S2 show the nutrient intakes provided by the standard formulations in comparison to the recommended nutrient intakes (RNI).

**Table 2:** Updated Amino acid-dextrose formulations

PN	Starter	Standard Preterm	High Sodium	7.5% Glucose Preterm	Peripheral preterm	34 weeks to Term
Indication	Birth to 24–48 h	After 24–48 h	Hyponatraemic Preterm	Hyperglycaemic Preterm	No central line	After 24–48 h
Concentration per litre						
AA, g	37.5	30	30	30	30	23
Dextrose, g	100	100	100	75	100	120
Na, mmol	20	34	60	34	34	25
K, mmol	0	22	22	22	22	20
Ca, mmol	17	17	17	17	3.5	7
Mg, mmol	1.5	1.5	1.5	1.5	1.5	1.5
P, mmol	10	13	13	13	3	4
Cl, mmol	10.1	12.7	30.7	12.7	18.7	28.2
Acetate, mmol	0	26	34	26	40	16.2
Zinc, μg	3270	3270	3270	3270	3270	1900
Selenium, μg	20	20	20	20	20	20
Iodine, μg	8.16	8.16	8.16	8.16	8.16	8.16
Heparin, units	500	500	500	500	500	500
Osmolarity, mosm	933	944	996	805	913	957
Nutrient intake when infused at 135 ml/kg/day						
AA, g		4.1	4.1	4.1	4.1	3.1
Dextrose, g		13.5	13.5	10.1	13.5	16.2
Na, mmol		4.6	8.1	4.6	4.6	3.4
K, mmol		3.0	3.0	3.0	3.0	2.7
Ca, mmol		2.3	2.3	2.3	0.5	0.9
Mg, mmol		0.2	0.2	0.2	0.2	0.2
P, mmol		1.8	1.8	1.8	0.4	0.5
Cl, mmol		1.7	4.1	1.7	2.5	3.8
Acetate, mmol		3.5	4.6	3.5	5.4	2.2
Zinc, μg		441	441	441	441	257
Selenium, μg		2.7	2.7	2.7	2.7	2.7
Iodine, μg		1.1	1.1	1.1	1.1	1.1

**Table 3:** New Starter Concentrated AA/dextrose formulation

	For preterm infants on restricted PN and water intake in the first 24–48 h. Not recommended at rate > 60 ml/kg/day					
	per 1000 mL	mL/kg/day				
		40	50	60	70	80
AA, g	50	2.0	2.5	3.0	3.5	4.0
Dextrose, g	100	4.0	5.0	6.0	7.0	8.0
Na, mmol	30	1.2	1.5	1.8	2.1	2.4
K, mmol	0	0.0	0.0	0.0	0.0	0.0
Ca, mmol	25	1.0	1.3	1.5	1.8	2.0
Mg, mmol	1.5	0.1	0.1	0.1	0.1	0.1
P, mmol	15	0.6	0.8	0.9	1.1	1.2
Cl, mmol	12.5	0.5	0.6	0.8	0.9	1.0
Acetate, mmol	0	0.0	0.0	0.0	0.0	0.0
Zinc, μg	3270	131	164	196	229	262
Selenium, μg	20	0.8	1.0	1.2	1.4	1.6
Iodine, μg	8.16	0.3	0.4	0.5	0.6	0.7
Heparin, units	500	20	25	30	35	40
Osmolarity, mosm/L	1069					
Solution pH	5.69	Alert - above maximal starter amino acid intake				
Bag volume, mL	500	Stability: up to 61 days @ 2-8 °C and 5 days at below 25 °C.

**Table 4:** New concentrated preterm AA/dextrose formulation

	For preterm infants with restricted PN or water intake after 24–48 h. Not recommended at rate > 100 ml/kg/day.								
	per 1000 mL	mL/kg/day							
		40	50	60	70	80	90	100	110
Amino acids, g	40	1.6	2.0	2.4	2.8	3.2	3.6	4.0	4.4
Dextrose, g	100	4.0	5.0	6.0	7.0	8.0	9.0	10.0	11.0
Na, mmol	50	2.0	2.5	3.0	3.5	4.0	4.5	5.0	5.5
K, mmol	35	1.4	1.8	2.1	2.5	2.8	3.2	3.5	3.9
Ca, mmol	22	0.9	1.1	1.3	1.5	1.8	2.0	2.2	2.4
Mg, mmol	1.5	0.1	0.1	0.1	0.1	0.1	0.1	0.2	0.2
P, mmol	15	0.6	0.8	0.9	1.1	1.2	1.4	1.5	1.7
Cl, mmol	39.6	1.6	2.0	2.4	2.8	3.2	3.6	4.0	4.4
Acetate, mmol	26	1.0	1.3	1.6	1.8	2.1	2.3	2.6	2.9
Zinc, μg	4900	196	245	294	343	392	441	490	539
Selenium, μg	30	1.2	1.5	1.8	2.1	2.4	2.7	3.0	3.3
Iodine, μg	12	0.5	0.6	0.7	0.8	1.0	1.1	1.2	1.3
Heparin, units	500	20	25	30	35	40	45	50	55
Osmolarity, mOsm/L	1092	Alert - below minimal recommended maintenance AA if no enteral intake							
Solution pH	5.75	Alert - above maximal recommended calcium and amino acid intake							
Bag volume, mL	750	Stability: up to 61 days @ 2-8 °C and 5 days at below 25 °C.

**Table 5:** Lipid/vitamin admixtures

SMOFLipid (Fresenius Kabi, Australia) formulations			
Contents	45 mL syringe For ≤1 Kg	90 mL bag - Unavailable	145 mL bag For > 1 Kg
SMOFlipid	32.5 mL		100 mL
Soluvit N	2.5 mL		8.4 mL
Vitalipid N Infant	10 mL		36.6 mL
ClinOleic (Baxter Healthcare, Australia) formulations			
Contents	45 mL syringe For ≤1 kg	90 mL bag For > 1 to ≤2 Kg	150 mL bag For > 2 kg
ClinOleic	32.5 mL	65 mL	108 mL
Soluvit N	2.5 mL	5 mL	8.4 mL
Vitalipid N Infant	10 mL	20 mL	33.6 mL

#### Cessation of PN

##### AA/dextrose infusion

There is no clear evidence to guide practice. The risk of late onset sepsis with intravenous access and the cost of PN are to be considered. The 2015 consensus survey found the majority of the NICUs in ANZ cease AA/dextrose formulation once the infant tolerates 120–140 ml/kg/day of enteral feeds [3].

##### Lipids

Mature human milk contains 3.4 g of fat per 100 mL. The 2015 consensus survey reported that the majority of NICUs cease IV lipids once the infant tolerates 100–120 mL/kg/day of enteral feeds [3].

#### Biochemical monitoring on PN

PN administration requires careful clinical and laboratory monitoring. High blood urea nitrogen, hyperglycaemia, metabolic acidosis, hypertriglyceridemia and conjugated hyperbilirubinemia are frequently encountered biochemical abnormalities on PN. In addition to routine observations, periodic measurements of the following biochemical parameters are suggested during PN therapy. Suggested monitoring by the group is shown in Additional file 1: Table S3.

No data are available to determine the effect of higher versus lower amino acid and lipid intake in PN in ‘sick’ infants (e.g., infants with moderate-severe respiratory distress, receiving cardiovascular support, possible sepsis, acidosis); and ‘surgical’ or postoperative infants or infants post-cardiopulmonary bypass.

**Blood Urea Nitrogen [**Conversion blood urea nitrogen = blood urea divided by 2.14]: In the Cochrane review comparing higher versus lower amino acid intake in parenteral nutrition, six studies reported BUN levels [9]. The criteria for abnormal blood urea nitrogen differed between the studies and varied from > 10 mmol/L to 21.4 mmol/L. There was a significant increase in abnormal blood urea nitrogen level from higher amino acid intake in all these studies although a threshold level was not clear. Given the data supporting the importance of early amino-acid administration in premature infants, limiting amino acid intake based on serum BUN alone is not warranted. BUN levels up to 14.3 mmol/L may be considered acceptable in VLBW infants on PN provided there are no other parameters to suggest protein intolerance (eg hyperammonaemia > 122 μmol/L) [9].

##### Hyperglycaemia

Hyperglycaemia (> 8.3 mmol/L) is not uncommon in ELBW infants [36]. If blood glucose > 10 mmol/L (moderate hyperglycaemia) [37, 38], further management to control hyperglycaemia should be considered including reducing the glucose infusion rate (e.g. changing over to 7.5% Dextrose PN).

**Hypoglycaemia** (BGL < 2.6 mmol/L) [39, 40] can occur particularly with a sudden cessation of PN or undetected extravasation of solutions.

##### Cholestasis

Defined as serum level of direct bilirubin > 20% of total serum bilirubin or serum level of direct bilirubin > 34 mmol/L [mg/dL × 17.10] [41].

##### Hypoalbuminemia

Defined as serum albumin, preterm < 18 g/L in preterm [42, 43] and < 25 g/L in term neonates [43].

##### Hypertriglyceridemia (HT) (plasma triglyceride > 2.8 mmol/L)

ESPGHAN 2005 Guidelines recommend monitoring of triglycerides in preterm and term infants and suggest a triglyceride level of 2.8 mmol/L as the upper limit [6]. The 2015 Consensus survey revealed 62% of respondents monitor plasma triglyceride levels either routinely or in specific circumstances [3]. A retrospective review from an Australian NICU in which routine triglyceride monitoring is in place showed HT incidence of 32.5% in 23–25 weeks and 16.1% in 26–28 weeks. Severe HT (> 4.5 mmol/L) was noted in 10% in 23–25 weeks and 4.5% in 26–28 weeks. HT was associated with a significant increase in mortality (unadjusted OR 3.5; 95% CI 1.13–10.76; 0.033) and severe retinopathy of prematurity (unadjusted OR 4.06; 95% CI 1.73–9.59; 0.002) on univariate analysis. Further multivariate analysis with adjustment for gestation and birthweight showed no significant association with HT [44].

#### Prolonged PN usage

The current formulations do not contain copper, manganese and molybdenum. Infants (e.g. post-surgical infants) who are exclusively on PN for long periods (> 4 weeks) may be at risk of trace element deficiency such as copper deficiency, which is associated with pancytopenia and osteoporosis [6]. These can be added to the current formulations for those infants on exclusive PN for more than 4 weeks [1].

#### PN in non-tertiary neonatal facilities

Many non-tertiary nurseries manage preterm and growth restricted term neonates, who may receive partial PN while establishing enteral feeds. As there is no clear evidence for this practice, the benefits of PN in this setting needs to be balanced against the potential risks of therapy, staff skill mix and the resource availability. Short term PN using peripheral preterm PN via peripheral cannula is appropriate for these infants if enteral feeding cannot be established by day 3–5 of life.

## Discussion

This updated consensus proposed a total of 8 AA/dextrose formulations, 3 ClinOleic lipid/vitamin admixtures and 2 SMOFlipid lipid/vitamin admixtures to cater for the need of the majority of the NICU population in the network. Recommendations are based on the majority consensus and mostly meet the recommended nutrient intakes when administered at recommended volumes. It is important to note that the 2017 Consensus Group considered recommendations from ESPGHAN guidelines published in 2005 [6]. Future meetings are planned to review and optimise these formulations, which will consider the more recent ESPGHAN guidelines published in 2018 [45]. While the number of formulations seems large, it is expected that individual units may choose to limit their formulations based on their current practice, size, the spectrum of their NICU population and preference. In view of the large number of formulations and significant improvement in the formulations from 2012, colour coded posters, handouts and worksheets have been developed to assist the frontline staff in the safe prescription of PN. Coloured labels have been developed for clear identification of each formulation. Most facilities in the Australian and New Zealand region procure PN formulations from the same commercial pharmaceutical companies. Formulations and guidelines to standardise parenteral nutrition practice across the Australasian region have the potential to improve nutrition and clinical outcomes of neonates. Standardisation of the formulations across Australia resulted in reduced practice variation and reduced labour costs in compounding the formulations with the pharmaceutical companies passing on the cost savings to the NICUs. Other potential benefits of standardisation, such as error minimisation in PN prescribing and ordering, will be investigated prospectively.

## Conclusions

The 2017 PN formulations and guidelines developed by the 2017 Neonatal Parenteral Nutrition Consensus Group offer concise and practical instructions to clinicians on how to implement current and up-to-date evidence based PN to the NICU population. In comparison to the previous formulations, the updated formulations have higher amino acid and other nutrients, particularly calcium and phosphate with the change from inorganic to organic phosphate. These PN solutions and guidelines have the potential to standardise the nutritional practice and improve both the quality and process of nutritional care and patient outcomes in the region.

## Supplementary information

**Additional file 1 Table S1.** 2017 consensus formulations and comparison to recommended parenteral nutrient intakes in preterm neonates. Values are per kg per day, unless otherwise indicated. **Table S2.** 2017 consensus formulations and comparison to recommended parenteral nutrient intakes in term neonates. Values are per kg per day, unless otherwise indicated. **Table S3.** Suggested routine PN biochemistry orders.

## Data Availability

Not applicable.

## Abbreviations

AA: Amino acids
AAP: American Academy of Pediatrics
CI: Confidence Interval
ELBW: Extremely Low Birth Weight
ESPGHAN: European Society of Paediatric Gastroenterology, Hepatology and Nutrition
GOR: Grade of recommendation
LBW: Low Birth Weight
LOE: Level of evidence
NICU: Neonatal intensive care unit
NSW: New South Wales
OR: Odds Ratio
PN: Parenteral Nutrition
RNI: Recommended Nutrient Intakes
RR: Relative Risk
