# Supplementary material for: Standardised neonatal parenteral nutrition formulations – Australasian neonatal parenteral nutrition consensus update 2017
Source: BMC Pediatr. 2020 Feb 8;20:59. doi: 10.1186/s12887-020-1958-9 (PMC7007668; doi:10.1186/s12887-020-1958-9)
Supplement: Supplementary file 1 — Additional file 1 Table S1. 2017 consensus formulations and comparison to recommended parenteral nutrient intakes in preterm neonates. Values are per kg per day, unless otherwise indicated. Table S2. 2017 consensus formulations and comparison to recommended parenteral nutrient intakes in term neonates. Values are per kg per day, unless otherwise indicated. Table S3. Suggested routine PN biochemistry orders. [file 12887_2020_1958_MOESM1_ESM.docx]

Table S1. 2017 consensus formulations and comparison to recommended parenteral nutrient intakes in preterm neonates. Values are per kg per day, unless otherwise indicated.

|  | | | | | | |
| --- | --- | --- | --- | --- | --- | --- |
| Nutrient | ESPGHAN 2005^6^ | | | AAP 2014 consensus^5^ | | 2017 consensus® |
|  | Preterm | | | Weight <1000 gm | Weight 1000-1500 g |  |
|  | Day 0 | Transition | Growing |  |  |  |
| Energy, Kcal |  |  |  | 105-115 | 90-100 | 100 |
| Protein, g | ≥1.5 | ≤4.0 | ≤4.0 | 3.5-4 g | 3.2-3.8 g | 4.05 g |
| Carbohydrate, g | 5.8-11.5 | ≤12.0 | ≤12.0 | 13-17 g | 9.7-15 g | 13.5 g |
| Fat, g | 1 | 1.0-3.0* | ≤3.0* | 3.0 - 4.0 | 3.0 - 4.0 | 3 |
| Linoleic acid, mg | ≥250 |  |  | 340-800 | 340-800 | - |
| Sodium, mmol | 0-3.0 (0-7d) | 2.0-3.0 | 3.0-5.0 | 3.0-5.0 | 3.0-5.0 | 4.59 |
| Potassium, mmol | 0-2.0 (0-7d) | 1.0-2.0 | 2.0-5.0 | 2.0 - 3.0 | 2.0 - 3.0 | 2.97 |
| Chloride, mmol | 0-5.0 (0-7d) | 2.0-3.0 |  | 3.0 - 7.0 | 3.0 - 7.0 | 1.7 |
| Calcium, mmol |  |  | 1.3-3.0 | 1.5-2.0 | 1.5-2.0 | 2.3 |
| Phosphate, mmol |  |  | 1.0-2.3 | 1.5-1.9 | 1.5-1.9 | 1.8 |
| Mg, mmol | 0.2 | 0.2 | 0.2 | 0.17-0.29 | 0.17-0.29 | 0.2 |
| Iron, µg | 0 | 0 (<3 wks) | 50-200 | 100-200 | 100-200 | - |
| Zinc, µg | 450-500 | 450-500 | 450-500 | 400 | 400 | 441 |
| Copper, µg | 20 | 20 | 20 | 20 | 20 | - |
| Selenium, µg | 2.0-3.0 | 2.0-3.0 | 2.0-3.0 | 1.5-4.5 | 1.5-4.5 | 2.7 µg |
| Chromium, µg | 0 | 0 | 0 | 0.05-0.3 | 0.05-0.3 | - |
| Molybdenum, µg | 1 | 1 | 1 | 0.25 | 0.25 | - |
| Manganese, µg | <1 | <1 | <1 | 1 | 1 | - |
| Iodine, µg/day | 1 | 1 | 1 | 1 | 1 | 1.1 µg |
| Vitamin A, IU | 495-990 | 495-990 | 495-990 | 700-1500 | 700-1500 | 920 |
| Vitamin D, IU | 32 | 32 | 32 | 40-160 | 40-160 | 160 |
| Vitamin E, IU | 2.8-3.5 | 2.8-3.5 | 2.8-3.5 | 2.8-3.5 | 2.8-3.5 | 2.8 |
| Vitamin K, µg | 10 | 10 | 10 | 10 | 10 | 80^#^ |
| Thiamin, µg | 350-500 | 350-500 | 350-500 | 200-350 | 200-350 | 310 |
| Riboflavin, µg | 150-200 | 150-200 | 150-200 | 150-200 | 150-200 | 360^#^ |
| Niacin, mg | 4.0-6.8 | 4.0-6.8 | 4.0-6.8 | 4.0-6.8 | 4.0-6.8 | 4 |
| Pyridoxine, µg | 150-200 | 150-200 | 150-200 | 150-200 | 150-200 | 400^#^ |
| Folate, µg | 56 | 56 | 56 | 56 | 56 | 40^*^ |
| Vitamin B12, µg | 0.3 | 0.3 | 0.3 | 0.3 | 0.3 | 0.5^#^ |
| Pantothenate, mg | 1.0-2.0 | 1.0-2.0 | 1.0-2.0 | 1.0-2.0 | 1.0-2.0 | 1.5 |
| Biotin, µg | 5.0-8.0 | 5.0-8.0 | 5.0-8.0 | 5.0-8.0 | 5.0-8.0 | 6 |
| Vitamin C, mg | 15-25 | 15-25 | 15-25 | 15-25 | 15-25 | 10^*^ |
| Acetate, mmol |  |  |  |  |  | 3.51 |

**®**At135ml/kg/d of standard Preterm PN plus 3 g/kg/d of Lipid: ^*^Below RDI, #Above RDI

Table S2. 2017 consensus formulations and comparison to recommended parenteral nutrient intakes in term neonates. Values are per kg per day, unless otherwise indicated.

| **Nutrient** | ESPGHAN 2005 | | | **2017 consensus®** |
| --- | --- | --- | --- | --- |
|  | Day 0 | | | ≤30 days |
| Energy, Kcal |  | 90-100 | 1-12 months |  |
| Protein, g | 1.5-3.0 | 1.5-3.0 | 90-100 | 104 |
| Carbohydrate, g | 5.8-11.5 | ≤18.0 | 1.0-2.5 | 3.1 |
| Fat, g | 1 | 3.0-4.0 | 5.0-12 | 16.2 |
| Sodium, mmol | 0-3.0 (0-7days) | 2.0-5.0 | 3.0-4.0 | 3 |
| Potassium, mmol | 0-2.0 (0-7days) | 1.0-3.0 | 2.0-3.0 | 3.4 |
| Chloride, mmol | 0-5.0 (0-7days) |  | 1.0-3.0 | 2.7 |
| Calcium, mmol | 0.8 | 0.8 |  | 2.7 |
| Phosphate, mmol | 0.5 | 0.5 |  | 2 |
| Magnesium, mmol | 0.2 | 0.2 |  | 1.6 |
| Iron, µmol | 0 | 0 (<3 weeks) | 0.2-0.3 | 0.2 |
| Zinc, µg | 250 | 250 | 1.8-3.6 |  |
| Copper, µg | 20 | 20 | 100 (>3 months) | 257 |
| Selenium, µg | 2.0-3.0 | 2.0-3.0 | 20 |  |
| Chromium, µg | 0 | 0 | 2.0-3.0 | 2.7 |
| Molybdenum, µg | 0.25 | 0.25 | 0 |  |
| Manganese, µg | <1 | <1 | 0.25 |  |
| Iodine, (µg/day) | 1 | 1 | <1 |  |
| Vitamin A, IU | 495-990 | 495-990 | 1 | 1.1 |
| Vitamin D, IU | 32 | 32 | 495-990 | 920 |
| Vitamin E, IU | 2.8-3.5 | 2.8-3.5 | 32 | 160 |
| Vitamin K, µg | 10 | 10 | 2.8-3.5 | 2.8 |
| Thiamin, µg | 350-500 | 350-500 | 10 | 80^#^ |
| Riboflavin, µg | 150-200 | 150-200 | 350-500 | 310 |
| Niacin, mg | 4.0-6.8 | 4.0-6.8 | 150-200 | 360^#^ |
| Pyridoxine, µg | 150-200 | 150-200 | 4.0-6.8 | 4 |
| Folate, µg | 56 | 56 | 150-200 | 400^#^ |
| Vitamin B12, µg | 0.3 | 0.3 | 56 | 40^*^ |
| Pantothenate, mg | 1.0-2.0 | 1.0-2.0 | 0.3 | 0.5^#^ |
| Biotin, µg | 5.0-8.0 | 5.0-8.0 | 1.0-2.0 | 1.5 |
| Vitamin C, mg | 15-25 | 15-25 | 5.0-8.0 | 6 |
|  |  |  |  |  |

®At135ml/kg/d of standard term PN plus 3 g/kg/d of Lipid: *Below RDI; #Above RDI.

Table S3. Suggested routine PN biochemistry orders

| **Test** | **First 3-7 days** | **Thereafter** |
| --- | --- | --- |
| Electrolytes, BUN, HCO3, Creatinine | Daily or as needed | Once or twice a week |
| Ca, PO4, Mg, bilirubin, albumin | As needed | Once a week |
| Triglyceride | 24 hours after each increase | Once a week |
| Blood glucose | 4-6 hourly | Once or twice a day |
| Liver function test including alkaline phosphatase | As needed | Once weekly or fortnightly |
